# Microbial network of the carbonate precipitation process induced by microbial consortia and the potential application to crack healing in concrete

**DOI:** 10.1038/s41598-017-15177-z

**Published:** 2017-11-03

**Authors:** Jiaguang Zhang, Aijuan Zhou, Yuanzhen Liu, Bowei Zhao, Yunbo Luan, Sufang Wang, Xiuping Yue, Zhu Li

**Affiliations:** 1 College of Architecture and Civil Engineering, Taiyuan University of Technology, Taiyuan, China; 2 Shanxi Construction Engineering Group Corporation, Taiyuan, China; 3 College of Environmental Science and Engineering, Taiyuan University of Technology, Taiyuan, China; 4 College of mechanics, Taiyuan University of Technology, Taiyuan, China

## Abstract

Current studies have employed various pure-cultures for improving concrete durability based on microbially induced carbonate precipitation (MICP). However, there have been very few reports concerned with microbial consortia, which could perform more complex tasks and be more robust in their resistance to environmental fluctuations. In this study, we constructed three microbial consortia that are capable of MICP under aerobic (AE), anaerobic (AN) and facultative anaerobic (FA) conditions. The results showed that AE consortia showed more positive effects on inorganic carbon conversion than AN and FA consortia. Pyrosequencing analysis showed that clear distinctions appeared in the community structure between different microbial consortia systems. Further investigation on microbial community networks revealed that the species in the three microbial consortia built thorough energetic and metabolic interaction networks regarding MICP, nitrate-reduction, bacterial endospores and fermentation communities. Crack-healing experiments showed that the selected cracks of the three consortia-based concrete specimens were almost completely healed in 28 days, which was consistent with the studies using pure cultures. Although the economic advantage might not be clear yet, this study highlights the potential implementation of microbial consortia on crack healing in concrete.

## Introduction

Calcium carbonate (CaCO_3_) precipitation is a common and circumstantial behavior for sequestration of large amounts of carbon on earth, which accounts for 41.9% of the total carbon^1^. Currently, microbially induced CaCO_3_ precipitation (MICP) has been regarded as an environmentally friendly and economical technique under different environmental conditions^2^. Boquet *et al*. showed that almost all kinds of bacteria can mediate the precipitation of CaCO_3_ if given suitable conditions^3^. This was mainly ascribed to their ability to create an alkaline environment through various metabolic activities, such as sulfate reduction, iron reduction, urea hydrolysis, denitrification, methane oxidation and photosynthesis^2^. Under alkaline conditions (pH > 8), CO_2_ in solution involved in the well-studied CO_2_–HCO_3_^−^–CO_3_^2−^ bicarbonate equilibrium could easily transform to CO_3_^2−^. Meanwhile, bacteria could also metabolize dissolved organic carbon into inorganic carbon (IC). Furthermore, bacterial cells act as ideal localized precipitation micro-environments for the formation of CaCO_3_ crystals^4^.

Extensive investigations of MICP were conducted for wide engineering applications in diverse biological, geological, and engineered systems, such as soil improvement^5^, metal remediation^6^, calcium-rich industrial wastewater treatment^7^, oil recovery^8^, the impacts on biofilms^9^, etc. More recently, calcium carbonate biomineralization has also been explored as a novel biotechnology for the restoration of construction materials, because biogenic CaCO_3_ possesses a favorable compatibility with the cementitious materials matrix^10^. With concerns about the high maintenance and repair costs of concrete, self-healing technology for the cracks in concrete based on MICP has become a research topic of great interest. The microorganisms involved in carbonate biomineralization cover nearly all classes^2^. However, concerning the high alkalinity in the concrete matrix, only alkali-tolerant and alkaliphilic strains can be used in a concrete environment. So far, aiming to strengthen the durability of concrete, the effect of various spore-forming and pure-culture bacteria on the crack-healing of concrete has been broadly discussed by many researchers^11^.

Up to now, the mechanism of MICP applied in the restoration of the durability of concrete can be classified into two categories. These are mainly based on urea hydrolysis by ureolytic bacteria and respiration by non-ureolytic bacteria. Urea hydrolysis could produce urease and harvest a significant amount of CO_3_^2−^, meanwhile, the increased pH in the surroundings leads to further accumulation of CaCO_3_ inside the cracks in concrete. *Bacillus sphaericus* and *Sporosarcina pasteurii*, two main types of ureolytic bacteria, were extensively investigated in these studies^12,13^. However, MICP through ureolysis has certain drawbacks, such as the secondary pollution introduced by the ammonia generated and the limited ureolysis efficiency caused by anaerobic/anoxic conditions. To prevent these issues, promising results have been obtained with non-ureolytic bacteria, i.e., *B*. *cohnii*, *B*. *pseudofirmus*, *B*. *subtilis* and *B*. *alkalinitrilicus*^14,15^. These bacteria can metabolize organic compounds (e.g., acetate and lactate), instead of urea, as the electron donor to induce the precipitation of CaCO_3_. Concrete durability was clearly improved by these pure cultures; nevertheless, there were very few reports concerned with microbial consortia, i.e., multiple interacting microbial populations, which can be more robust in their resistance to environmental fluctuations, perform more complicated tasks and endure more changeable environments than individual populations can^16^.

Therefore, based on the abovementioned considerations, three microbial consortia that are capable of carbonate precipitation were constructed under aerobic (AE), anaerobic (AN) and facultative anaerobic (FA) conditions. Given that CaCO_3_ precipitation is a rather straightforward chemical process governed by the pH and dissolved IC, we monitored the dissolved IC concentration and pH value during the selection of microbial consortia. To investigate the efficiency of CaCO_3_ precipitation introduced by the three microbial consortia, potential application to crack healing in concrete was studied. Furthermore, we also examined the microbial community structure, using high-throughput pyrosequencing of the small subunit ribosomal ribonucleic acid (RNA) (16S rRNA) gene, which can provide important information to better understand the microbial response mechanism to the three microbial consortia.

## Materials and Methods

### Selection of microbial consortia capable of carbonate precipitation

Three enrichment cultures were established with activated sludge (Jinzhong municipal wastewater treatment plant, Taiyuan City, China) and garden soil (Taiyuan University of Technology (Mingxiang Campus), Taiyuan City, China) as microbial sources under AE, AN and FA conditions. Cell suspensions were prepared by adding 10 g of garden soil and 10 mL of activated sludge to 500 mL flasks containing 200 mL Luria-Bertani medium (per liter: 10 g of tryptone, 5 g of yeast extract, 10 g of NaCl) as a germinant to induce the germination of dormant bacteria. All flasks were shaken for 30 min at 150 rpm at 30 ± 2 °C. To start the selection experiments of AE microbial consortia, aliquots (10 mL) of cell suspension were added to triplicate 300 mL Erlenmeyer flasks containing 100 mL of aerobic-selection medium (AE_SM, 500 mg/L sodium lactate (C_3_H_5_O_3_Na) (as chemical oxygen demand (COD)), 20 mg/L NH_4_Cl, pH 11.0) with 1% of Wolf’s trace mineral and vitamin solutions^17^. Flasks were incubated at 150 rpm at 30 ± 2 °C under aerobic conditions. Once the bacterial cell density of selection systems reached approximately 5 log cells/mL (approximately 2 days), aliquots (10 mL) of microbial culture were transferred to fresh medium (diluted 1000 times). These procedures were repeated nine times. Part of the bred samples were taken from each microbial consortium at transfer 10 and stored with 20% of glycerol at −80 °C. For the selection of the AN microbial consortia, anaerobic-selection medium (AN_SM, 500 mg/L (as COD) C_3_H_5_O_3_Na, 30 mg/L NaNO_3_, pH 11.0) was used. The cultivation was conducted in an incubator at 30 ± 2 °C for approximately 3 days. For the selection of FA microbial consortia, the AN microbial consortia at transfer 5 were introduced into fresh AE_SM medium, and the bred consortia were subsequently transferred to fresh AN_SM medium. These procedures were repeated five times. All other operations were the same as described above. The consortia of the flasks at transfer 10 under AE, AN and FA conditions were used for the subsequent analyses (hereinafter referred to as the AE, AN and FA tests or samples).

### Experiment setup procedure of cracks healing in concrete

Concrete specimens were prepared as previously described^18^. To immobilize the bacteria, expanded perlite (EP) particles were impregnated under vacuum with the three prepared microbial consortia suspensions and dried in an oven to constant weight at 45 °C for 2 days. The cell density of 5 log cells/mL was used to prepare all the bacteria-based concrete mixtures. A solution including calcium lactate (8 g/L) and yeast extract (1 g/L) was sprayed on the surface of the particles with immobilized AE microbial consortia. For the particles with immobilized AN and FA microbial consortia, 4 g/L calcium lactate, 4 g/L calcium nitrate and 1 g/L yeast extract were sprayed. After the spraying and coating of the EP particles^18^, prismatic specimens with dimensions of 15 × 15 × 30 cm were cast for each mix, named “AE”, “AN” and “FA”, respectively. No microbial consortia were added in the control mix (named “Control”). After 28 days of curing, all the specimens were pre-cracked using an electro-hydraulic servo testing machine. The widths of the selected cracks varied from 0.1 mm to 0.9 mm. Then, the pre-cracked specimens were marked and immersed horizontally in tap water in three plastic buckets for crack healing. All the buckets were kept open to the atmosphere at standard room temperature (20 ± 2 °C). It needs be mentioned that aeration was provided to increase the oxygen supply for the bucket, in which the specimen with immobilized AE microbial consortia was immersed. The dissolved oxygen concentration was above 2.0 mg O_2_/L. All the specimens were taken out of water for microscopic inspection and crack width measurement after 7 and 28 days healing respectively. Quantification of crack-healing in these specimens was conducted in time to investigate the crack-healing capacity of different bacterial self-healing systems.

### DNA extraction and pyrosequencing

Before DNA extraction, three selected microbial consortia were centrifuged at 8000 g to remove supernatant. DNA was extracted from the sediments of three replicate flasks using a EZNA^®^ Soil DNA kit (Omega Bio-Tek, Inc., Norcross, GA, USA) and then pooled together. Amplicon liberates were constructed for pyrosequencing using bacterial fused primers 341 F and 805 R for the V3-V4 region of the 16S rRNA gene^19^. A common way to distinguish the products of a multiplex genotyping reaction is to incorporate specific nucleotide sequences, i.e., “barcodes”, into the allele-specific genotyping oligos. To achieve the sample multiplexing during pyrosequencing, barcodes were incorporated between the 454 adaptor and the forward primer. The 454 adaptor was the amplicon sequencing primer annealing site. Polymerase chain reactions (PCRs) were performed according to our previous studies^20,21^. After being purified and quantified, the PCR amplicon was used for pyrosequencing on an Illumina MiSeq. The raw sequences were deposited in the NCBI Short Read Archive database with the accession no. SRR5456943. The adaptors, barcodes, and primers in all raw sequences were trimmed to minimize the effects of random sequencing errors. Sequences shorter than 350 bp, or containing any ambiguous base calls, were removed. The remaining sequences were clustered into operational taxonomic units (OTUs), using the 97% identity threshold (3% dissimilarity level). Rarefaction curves were generated and alpha diversity measurements, including the Shannon index (http://www.mothur.org/wiki/Shannon), the Chao1 index (http://www.mothur.org/wiki/Chao) and the ACE index (http://www.mothur.org/wiki/Ace), were calculated for each sample. OTUs networks were visualized in Cytoscape v3.2.1 for depicting the similarity and difference between the different microbial consortia^22^.

### Analytical methods

Microbial consortia samples were centrifuged at 10,000 g for 10 min, filtered through a 0.45 μm cellulose nitrate membrane filter and stored at 4 °C prior to analysis. The contents of total organic carbon (TOC) and inorganic carbon (IC) were measured by a TOC analyzer (TOC-VCPH, Shimadzu, Japan). The IC conversion rate (IC-CR) is calculated by the following equation:

1 $${\rm{IC}} \mbox{-} {\rm{CR}}\,( \% )=({{\rm{IC}}}_{t}\text{-}{{\rm{IC}}}_{0})/{{\rm{TOC}}}_{0}\times 100 \% $$

where IC_*t*_ is the IC concentration at time t (h), IC_0_ is the initial IC concentration in the selection medium, and TOC_0_ is the initial TOC concentration in the selection medium. The pH value was measured by a pH meter (Seven Multi, Mettler Toledo, Switzerland). The crack widths were measured along the length of each crack at regular intervals (every 1 cm). The percentage of crack healing at each location on each specimen was calculated as follows^23^:

2 $${\rm{Healing}}\,{\rm{percentage}}\,( \% )=({d}_{0}\,-\,{d}_{t})/{d}_{0}\times 100$$

where *d*_0_ is the initial crack width, *d*_t_ is the width measured at healing time *t*. Thirty measurements covering distinct cracks at different healing times were conducted in each type of specimens.

## Results and Discussion

### Time-courses of IC conversion using three selected microbial consortia

**Figure 1:**
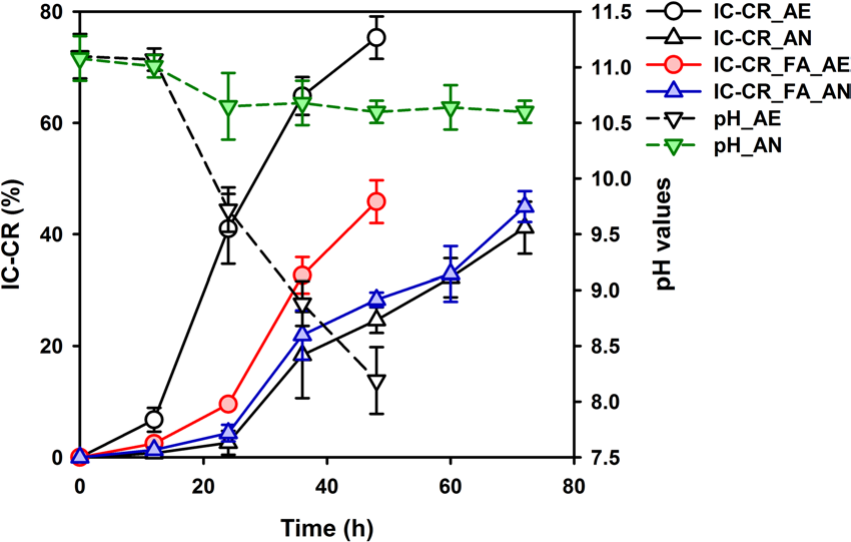
The variation of pH and IC-CR values in the presence of different microbial consortia (Note: error bars represent the standard deviation).

The time-course profiles of pH values and IC-CR rates in three selected microbial consortia are shown in Fig. 1. The result disclosed that all these three selected microbial consortia had a positive effect on the IC conversion from organic matter but their extent varied. A marked rise for IC-CR in the AE test (75.3 ± 3.8%) was observed during 48 h of cultivation, with an increase of up to 1.2~1.8 times compared with the other three tests (AN, FA_AE and FA_AN). These results were further supported by a variation in pH. As depicted in Fig. 1, the pH values of the AE test sharply decreased with the increase in cultivation time. By contrast, the reduction of pH values was relatively slow for the AN test, from approximately 11.0 to 10.5. The reason was presumably that the concentration of IC related to metabolic utilization of dissolved CO_2_, which would induce a shift in the bicarbonate/carbonate equilibrium and a subsequent pH reduction in the bulk medium (Equations 3)^10^.

3 $${{\rm{CO}}}_{2}+{{\rm{H}}}_{2}{\rm{O}}\leftrightarrow {{{\rm{HCO}}}_{3}}^{-}+{{\rm{H}}}^{+}\leftrightarrow {{{\rm{CO}}}_{3}}^{2-}+2{{\rm{H}}}^{+}$$

What is noteworthy is that the pH value of the AE sample at 48 h was 8.2 ± 0.3%. Boquet *et al*. showed that any increase in pH (above pH 8.0) generated as a result of bacterial metabolism can lead to carbonate precipitates^3^. That is, CaCO_3_ precipitation could occur if soluble calcium ions are present under the AE conditions, as it is for the AN and FA tests.

### Microbial community distribution and diversity analysis

**Figure 2:**
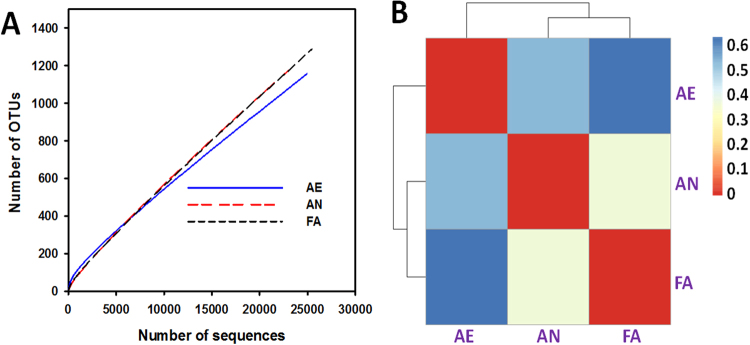
Rarefaction curves (**A**) and distance heatmap analyses (**B**) of bacterial communities from the three selected microbial consortia based on pyrosequencing of the 16S rRNA gene.

**Figure 3:**
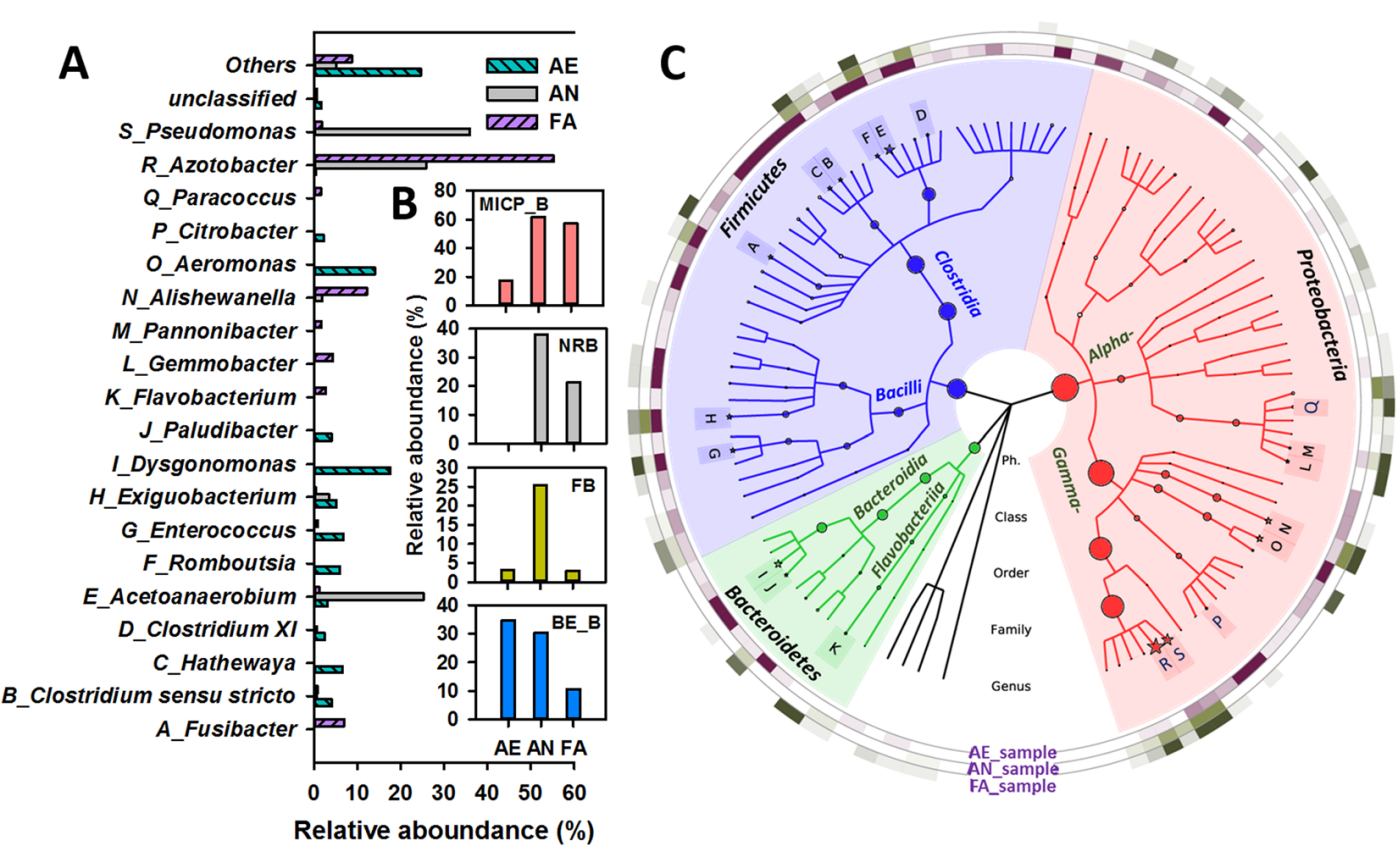
Taxonomic classification of pyrosequences from bacterial communities of three samples at the genus levels representing all OTUs present at a relative abundance > 1% (**A**). Relative abundance of the specific functional genera classification for MICP, DNB, BE and fermentation (**B**). Hierarchical clustering analysis of the bacterial communities of three samples (**C**).

To understand the mechanisms of carbonate precipitation through microbial activities, high-throughput pyrosequencing was conducted to evaluate the microbial diversity and distribution. Three 16S rRNA gene libraries were constructed from MiSeq sequencing, in total, with 74,132 of high-quality reads (average length of 420 bp), and they were subsequently clustered into 3,651 OTUs at a 3% distance (Table 1). Rarefaction curves for all libraries displayed shapes indicative of effective sampling of community diversity (Fig. 2A). The microbial diversities of the evolving communities were assessed based on α-diversity. The Shannon diversity index provided the species evenness, indicating that the AE sample showed the highest diversity (Shannon 3.59) among three communities. Based on the Chao1 and ACE indices, indicating the richness, the FA sample had a relatively lower diversity (19,193 and 68,621). In other words, a reduction in bacterial diversity occurred after the further selection of AN microbial consortia (Table 1). Distance heatmap (Fig. 2B) and hierarchical clustering analyses (Fig. 3C) were conducted to further illustrate the distribution and the similarity of the microbial communities. The AN and FA were clustered together. The AE microbial community was distinct from that of the AN and FA samples, suggesting clear distinctions in the community structure between different microbial consortia systems despite the fact that the same initial source of microbial consortia was shared. These results showed that particular bacteria were selectively enriched, and the different selection conditions had an obvious effect on the community structures.

**Table 1:** Alpha diversity of the three samples.

Name	Seq num^*1^	OTU num^*2^	Barcode	Shannon index	Chao1 index	ACE index	Coverage
AE	24945	1157	GTATCT	3.59	13371	38793	0.96
AN	23716	1205	AGGCGG	2.31	12060	32569	0.96
FA	25471	1289	TCTATT	2.34	19193	68621	0.95

^*1^“Seq num” indicated the sequence numbers obtained from the high-throughput sequencing analysis;

^*2^“OTU num” indicated the classified OTU numbers obtained from the gene sequences with the identity of over 97%.

The distribution of the microbial community was subsequently identified at the phylum, class and genus levels. Clear changes were observed in the microbial community structure after different selections. *Bacteroidetes*, *Firmicutes* and *Proteobacteria* were the dominant phyla for all three communities (accounting for ~99.8% of the total bacterial sequences). *Firmicutes* were primarily dominant in the AE sample (49.8%), compared with 32.1% and 11.6% in the AN and FA samples. Some species of *Firmicutes* were shown to play important roles in carbon recycling^24^. Almost nearly identical trend was observed for *Bacteroidetes*. Conversely, the proportion of *Proteobacteria* increased to a relatively higher value under AN and FA selections (67.4% and 84.4% respectively versus 25.4% in the AE sample). At the class level, seven major classes included the majority of the sequences. Among them, *Gamma-proteobacteria* (phylum *Proteobacteria*) were the dominant ones in the AN (65.6%) and FA (72.3%) samples, which are often considered r-strategists, i.e., with good dispersal and colonizing ability^25^, and should dominate when nutrients are plentiful^26^. *Bacteroidia* (phylum *Bacteroidetes*), as one of the few types of bacteria resistant to extreme pH conditions^27^, were evidently enhanced by the selection of the AE conditions, which made them capable of rapidly exploiting a new environment under harsh conditions. Interestingly, *Clostridia*, as obligate anaerobes, were also increased in the AE microbial consortia. This pyrosequencing result somewhat conflicted with known facts. The reason behind this may be that (1) the selection the microbial consortia only transferred 10 times. The number of cycles was relatively short for the bacteria selection; (2) a cotton plug was put in each of the shake flasks to transfer oxygen. Previous studies showed that a cotton plug in the shake flask can limit the mass transfer significantly such that the oxygen in the headspace may decrease and carbon dioxide may accumulate^28,29^. This may introduce anaerobic micro-organisms into the AE microbial consortia. Further studies will be performed on the improvement of the selection procedure to ensure a stable and effective microbial consortia.

**Figure 4:**
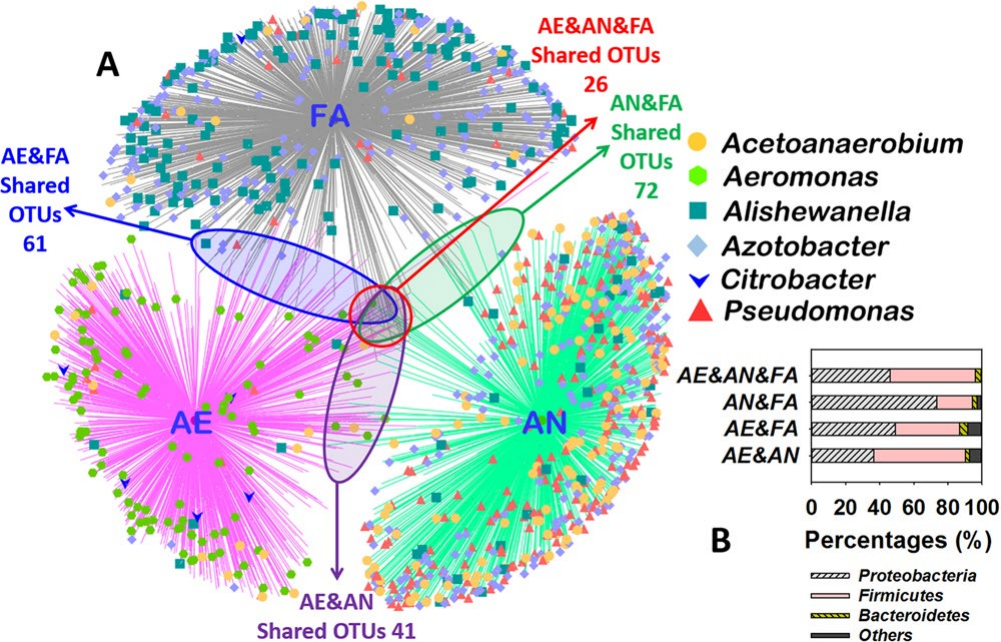
Network of communities based on OTUs in three selected microbial consortia (**A**). Overlap of the four bacterial communities based on OTU (3% distance) (**B**).

Further investigation on the genus level provided more detailed information about microbial communities (Fig. 4A). *Aeromonas* and *Citrobacter* (belonging to the class *Gamma-proteobacteria*) took up the largest proportion in the AE sample (14.1% and 2.3%), which were capable of forming CaCO_3_ under specific conditions^3,30^. As one of the class *Bacteroidia*, *Dysgonomonas* reached the highest abundance in AE (17.6%), which proved that it could transfer electrons from solution and predominate under conditions of high alkalinity^31,32^. It has been reported that more than 200 soil bacteria, including *Pseudomonas* spp. and *Azotobacter* spp., are capable of inducing CaCO_3_ precipitation^3^. The latter two were abundant in the AN (35.8% and 25.9%) and FA (1.9% and 55.2%) samples, respectively. That is, the proportion of *Azotobacter* was clearly increased after the facultative selection of AN microbial consortia. The other dominant genus in AN was *Acetobacterium* (Class *Clostridia*, 25.3%), which is specialized as an acetogenic bacterium^33^.

### Microbial community network on the specific functional genera

To elucidate the interactions among all the OTUs and analyze the shared and most abundant OTUs in the three microbial consortia samples, a network representing the community change and linkage was constructed (Fig. 4). Collectively, only 26 phylum-level OTUs were shared in these three samples. The number of OTUs shared by the AN and FA samples was 72, while 41 and 61 were shared by the AE&AN and AE&FA samples, respectively (Fig. 4A). Meanwhile, the majority of the shared OTUs were all *Proteobacteria* (46.2%~73.6%), *Firmicutes* (20.8%~53.7%) and *Bacteroidetes* (2.3%~4.9%) (Fig. 4B). The bacteria capable of carbonate precipitation (MICP_B), i.e., *Aeromonas* and *Citrobacter* (clearly enriched in the AE sample), *Pseudomonas* and *Azotobacter* (dominating the AN and FA samples) (Fig. 4A), accounted for approximately 17.0%, 61.7% and 57.1% of the population under the AE, AN and FA selections, respectively (Fig. 3B). Although the percentage of reported MICP_B in the AE sample was lower than that in the other samples, organic matter could be rapidly degraded by the AE microbial consortia to CO_2_ under the aerobic conditions. As mentioned above, this converted inorganic matter should easily precipitate in the presence of calcium ion in an alkaline environment. This study presents MICP through denitrification under anaerobic and facultative-anaerobic conditions; thus, nitrate-reducing bacteria (NRB) played a critical role in the AN and FA systems. *Pseudomonas* was also a common group of NRB that can convert NO_3_^−^ to N_2_ or NO_2_^−^ ^34^. Li *et al*. (2016) stated that most of the species of *Paracoccus* could use NO_3_^−^ as an electron acceptor alternative to oxygen with the N_2_ as final reduction product^35^. Previous studies showed that *Alishewanella*, *Flavobacterium*, *Enterococcus* and *Pannonibacter* could also use multiple electron acceptors for reducing NO_3_^−^ ^36,37^. That is, the sum of NRB accounted for 38.1% and 21.3% in the AN and FA samples, respectively. Specifically, as shown in Fig. 4A, *Alishewanella* (1.9% versus 12.2%) and *Pseudomonas* (35.8% versus 1.9%) were the dominant NRB in the AN and FA systems. Meanwhile, some fermentative bacteria, such as *Acetobacterium* and *Gemmobacter*, contributed to the metabolism of large molecular carbon source (lactic acid) to small molecular carbon sources (acetic acid, HAc), which peaked at 25.4% in the AN sample (Fig. 3B). Bacterial endospores (BE_B) constitute a survival strategy. More specifically the *Clostridia* and *Bacilli* classes can exhibit remarkable resistance to numerous environmental insults such as heat, desiccation, and extremes in pH and pressure^38^. The sum of BE_B peaked at 34.7% in the AE sample, followed by 30.4% in the AN sample.

**Figure 5:**
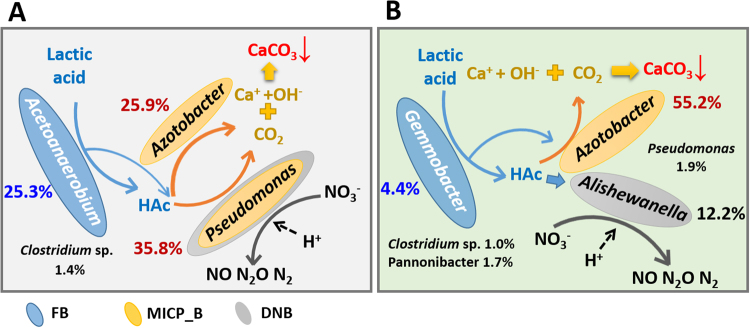
Observed interactions between key populations during MICP introduced by the AN and FA microbial consortia.

Based on the above discussion, the substrates and electron transfer of MICP through denitrification in the AN and FA systems could be explored (Fig. 5). The original rationale for realizing carbonate precipitation in the presence of nitrate was based on the highly negative standard Gibbs free energy (*ΔG*^*0′*^, −785 kJ/mol). The involved specific anaerobic reactions (4~7) were presented as follows:

4 $$3\,{\rm{Lactate}}={\rm{HAc}}+2\,{\rm{Propionate}}\,({\rm{HPr}})+{{{\rm{HCO}}}_{3}}^{-}+{{\rm{H}}}^{+}$$

5 $${\rm{HPr}}+3\,{{\rm{H}}}_{2}{\rm{O}}={\rm{HAc}}+{{{\rm{HCO}}}_{3}}^{-}+{{\rm{H}}}^{+}+3\,{{\rm{H}}}_{2}$$

6 $${{\rm{Ac}}}^{-}+2.6\,{{\rm{H}}}^{+}+1.6{{{\rm{NO}}}_{3}}^{-}=2{{\rm{CO}}}_{2}+0.8{{\rm{N}}}_{2}+2.8\,{{\rm{H}}}_{2}{\rm{O}}$$

7 $${{\rm{Ca}}}^{+}+{{\rm{CO}}}_{2}+2\,{{\rm{OH}}}^{-}={{\rm{CaCO}}}_{3}+{{\rm{H}}}_{2}{\rm{O}}$$

The result indicated that in the presence of *Acetoanaerobium*, *Clostridium* sp. and other anaerobic fermentation bacteria, lactate was metabolized into HAc, which was used as an electron donor for the identified microorganism (*Azotobacter* and *Pseudomonas*) involved in the MICP and denitrification processes in the AN system (Fig. 5A). This partnership had its advantages, e.g., *Pseudomonas* could precipitate carbonate in the MICP and denitrification processes simultaneously. Consistent with this partnership, and with NO_3_^−^ as the electron acceptor, HAc produced by *Gemmobacter*, *Pannonibacter*, *Clostridium* sp. and other facultative fermentation bacteria was further metabolized into inorganic carbon (CO_2_, HCO_3_^−^, etc). During this process, *Azotobacter* and *Alishewanella* acted as the key microorganisms involved in the MICP and denitrification processes in the FA system (Fig. 5B).

### Potential implementation on cracks healing in concrete

**Figure 6:**
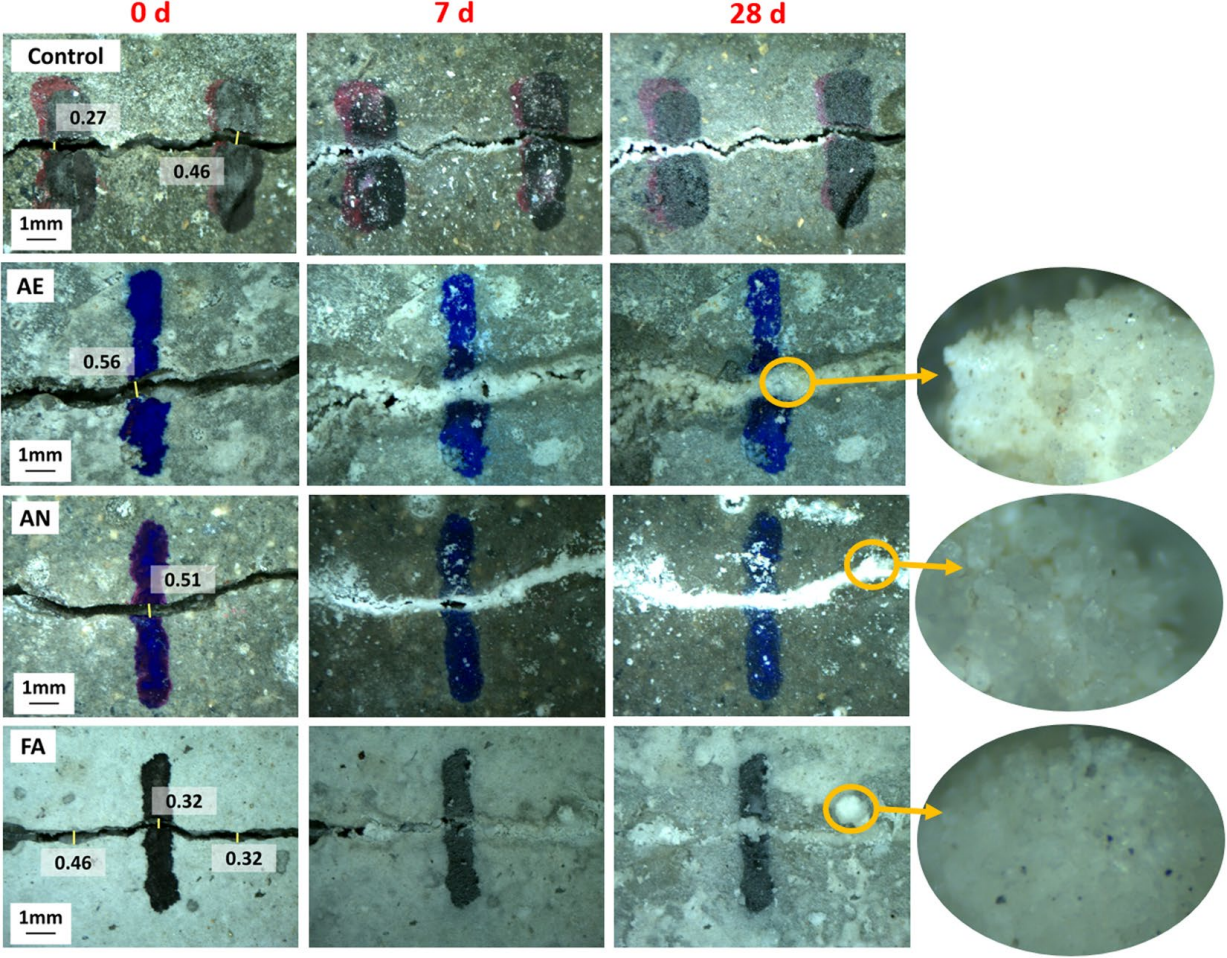
Microscopic images of four types of crack-healing processes.

To evaluate the MICP performance of the selected microbial consortia, three types of concrete specimens, immobilized AE, AN and FA microbial consortia, were prepared to quantify their crack healing. Figure 6 shows the microscopic images of cracks on the surfaces of the four types of specimens at different healing times. The opening of the crack is well defined at the beginning of crack healing (0 h). As time progressed, the crack widths of the bacteria-based concrete specimens were observed to gradually decrease. After 28 days of healing, the selected cracks of the three bacteria-based concrete specimens were almost completely healed (the crack widths were 0.32~0.56 mm), while those of the control specimen were barely healed (the crack width was 0.27 and 0.46 mm). A beige deposit could be visibly observed at the surface of the bacteria-based specimens, which was consistent with studies using pure cultures^15,23,39^.

**Figure 7:**
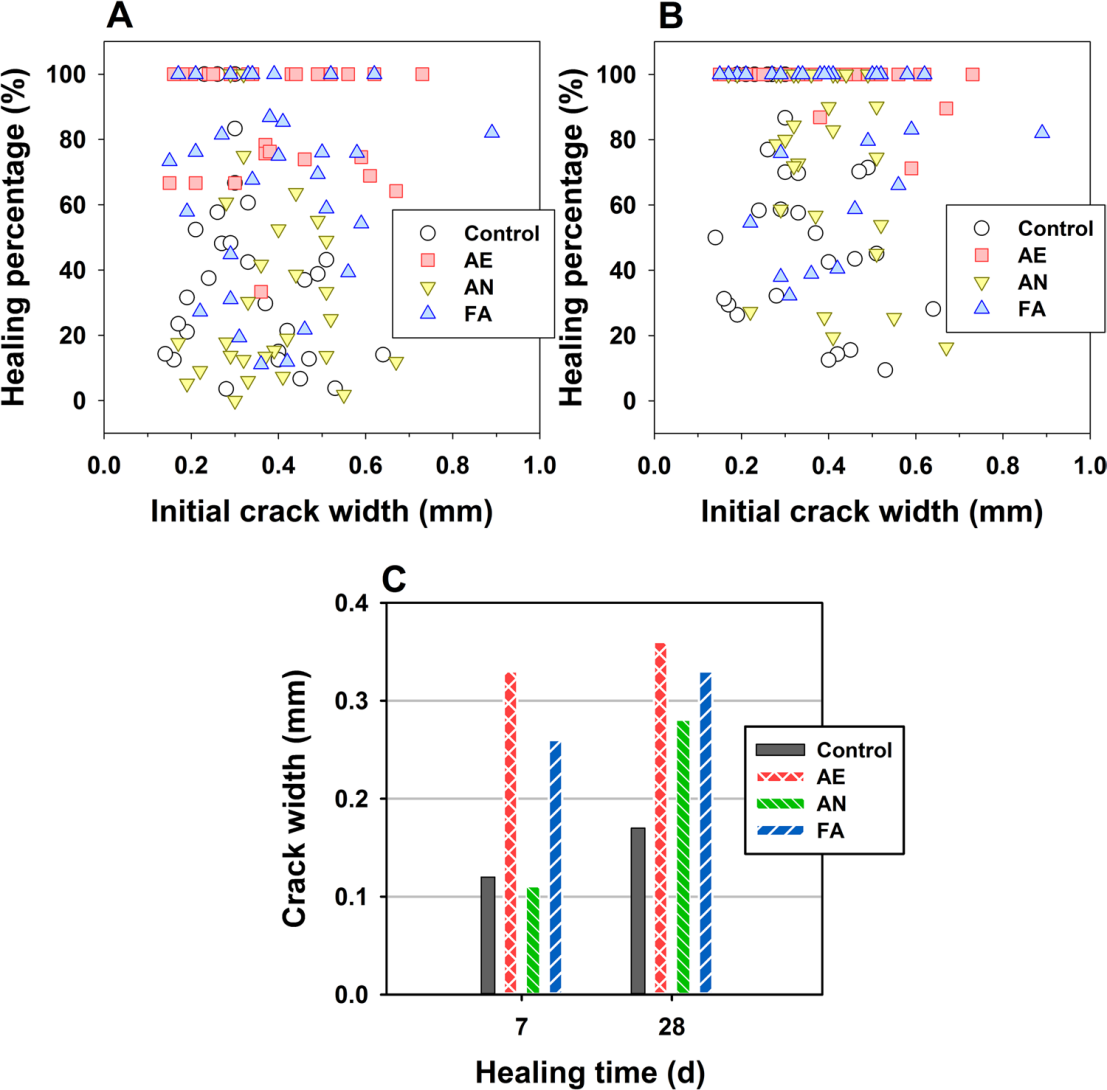
Crack healing percentage as a function of the initial crack width for concrete specimens at healing times of 7 days (**A**) and 28 days (**B**). Average values of healed crack widths at different healing times (**C**).

As shown in Fig. 7A, a higher crack-healing percentage was observed for the smaller crack width at a healing time of 7 d. The crack-healing percentages increased with the increase in healing time (Fig. 7B). Furthermore, it can be observed that the specimens subjected to the AE microbial consortia had a higher healing ability and faster healing rate, followed by the FA and AN tests. The average value of healed crack widths reached 0.36 mm in the AE specimens after 28 days of healing, while the values observed in the FA and AN specimens were 0.28 and 0.33 mm, respectively (Fig. 7C). The values changed slightly with the increase in healing time (from 7 d to 28 d) in the AE and FA specimens. In contrast, that increased gradually from 0.11 mm (7 d) to 0.28 mm (28 d) in the AN specimens. The results were somewhat in accordance with the experiment of IC conversion. The reason behind this maybe that this study provided feasible biological environments for AE microbial consortia, by external aeration to increase the oxygen supply, while the dissolved oxygen in tap water could affect the activity of the AN microbial consortia to some extent. In contrast, this environment was not harsh enough for the FA microbial consortia. Therefore, further studies should be performed on the improvement of healing regimes for concrete, with immobilized AN and FA microbial consortia, such as exposure to wet-dry cycles^40^. Since microbial consortia can perform more complex tasks and survive in more changeable environments than can uniform populations, the implementation of microbial consortia on crack healing in concrete will be of increasing interest. Certainly, the practical implementation should also further assess the potential challenges in the concrete structures associated with further management.

## Conclusions

This study constructed three microbial consortia under three conditions (AE, AN and FA) and studied the selected microbial consortia on the performance of IC conversion and suggested crucial implications for crack healing in concrete. A comprehensive study to shed light on the underlying mechanism was undertaken by means of process assessment associated with microbial community analysis. AE samples exerted a positive effect on the IC conversion (75.3 ± 3.8%), which was 1.2~1.8 times that of the other consortia. The overall analysis of pyrosequencing suggested that particular bacteria were selectively enriched in different microbial consortia and the different selection conditions had an obvious effect on the community structures. It is worth noting that species in the constructed microbial consortia could build complex networks of interaction, regarding MICP_B, NRB, BE_B and FB communities, by microbial community network analysis. Further investigation revealed that the implementation of microbial consortia on crack healing in concrete will be of increasing interest. Specimens immobilized AE microbial consortia exhibited better healing performance of concrete cracks.
