# Publisher Correction: Microbial network of the carbonate precipitation process induced by microbial consortia and the potential application to crack healing in concrete

**DOI:** 10.1038/s41598-018-19649-8

**Published:** 2018-01-15

**Authors:** Jiaguang Zhang, Aijuan Zhou, Yuanzhen Liu, Bowei Zhao, Yunbo Luan, Sufang Wang, Xiuping Yue, Zhu Li

**Affiliations:** 1 College of Architecture and Civil Engineering, Taiyuan University of Technology, Taiyuan, China; 2 Shanxi Construction Engineering Group Corporation, Taiyuan, China; 3 College of Environmental Science and Engineering, Taiyuan University of Technology, Taiyuan, China; 4 College of mechanics, Taiyuan University of Technology, Taiyuan, China

Correction to: *Scientific Reports* 10.1038/s41598-017-15177-z, published online 03 November 2017

The original HTML version of this Article contained a typographical error in the publication date ‘03 November 2017’ which was incorrectly given as ‘06 November 2017’. This has now been corrected in the HTML version of the Article.

